# A Large Stomach Ulcer Is Associated With Raised Mortality in a Cohort of Patients Who Underwent Open Repair of Perforated Peptic Ulcer: A Five-Year Follow-Up Study

**DOI:** 10.7759/cureus.9790

**Published:** 2020-08-16

**Authors:** Murad Aljiffry, Esraa A Alshehrani, Afnan Saeed, Fatemah Albugmi, Israa Alsulami, Walaa Alzahrani, Osman O Al-Radi, Anas H Alzahrani

**Affiliations:** 1 Surgery, King Abdulaziz University, Jeddah, SAU; 2 Surgery, King Abdulaziz University Hospital, Jeddah, SAU

**Keywords:** retrospective, perforated peptic ulcer, size, stomach, duodenum

## Abstract

Introduction

Perforated peptic ulcer disease (PPUD) is associated with a high postoperative mortality and morbidity rates especially within the first 90 days. The size and site of the ulcer may contribute to the prognosis of PPUD. In this study, we will describe the association of size and site of PPUD with the overall mortality and in-hospital morbidities in a tertiary care university hospital.

Methods

A retrospective observational cohort study was conducted at King Abdulaziz University Hospital, Jeddah, Saudi Arabia. A total of 50 patients who had PPUD and underwent open exploratory laparotomy with surgical treatment were analyzed. Patients were divided into two groups: a small ulcer group when the ulcer diameter was less than equal to 1 cm and a large ulcer group when it was more than 1 cm. For the subgroup analysis, patients were categorized according to site into small duodenum, large duodenum, small stomach, and large stomach PPUD. The primary outcome was overall mortality that was measured by survival analysis and Cox regression. Secondary outcomes were intensive care unit (ICU) admission, ICU and hospital length of stay, and in-hospital mortality, which were assessed by stepwise logistics and linear regression.

Results

Overall mortality at 10, 30, and 90 days was 14% (95% CI: 0.06-0.27), 24% (95% CI: 0.14-0.39), and 34% (95% CI: 0.23-0.49), respectively. Saudi patients had a 72% decreased risk of overall mortality compared to non-Saudi patients (P=0.03) over the follow-up period. Overall, patients who had stomach PPUD had a 2.23-fold increased risk of overall mortality over time compared to those who had duodenum PPUD (P=0.10). Large PPUD, >1 cm, had a 3.20-fold increased risk of overall mortality over time compared to small PPUD (P=0.04). Large stomach PPUD had a 4.22-fold increased risk of overall mortality over time compared to other ulcers (P=0.01).

Conclusions

Large stomach PPUD is associated with increased overall mortality and morbidity. These findings indicate that patients who have a large stomach PPUD might need careful perioperative and postoperative personalized surgical plans as these patients may eventually undergo complicated surgical procedures.

## Introduction

Perforation is a severe complication of peptic ulcer disease (PUD) and it occurs in 2% to 14% of patients with PUD [1]. Perforated PUD (PPUD) carries a risk of high mortality and morbidity, estimated to be 30% and 50%, respectively, within the first 30 days post-repair [2]. Several risk factors have a potential role in developing PPUD, including cigarette smoking, NSAID (nonsteroidal anti-inflammatory drug) use, Helicobacter pylori infection, and a history of previous PUD [1]. The postoperative complications are mainly related to patients’ factors rather than in-hospital care [3]. However, these risk factors and their relation to morbidity and mortality rates are not well described in the literature.

The size of the ulcer is one of the important factors as patients usually develop <0.5 cm perforation without significant mucosal defect, whereas others develop >1cm perforation with a significant mucosal defect [4]. The larger the size of PPUD (>0.5 cm) together with multiple gut perforations were related to a poor prognosis with overall mortality of 32% in 30 days [5]. With regard to the site, the incidence of perforated duodenal ulcer has increased in the last decade compared to perforated stomach ulcers particularly in females [5].

We hypothesize that the size and site and of PPUD may play important roles in patients’ overall mortality and in-hospital morbidity. The main objectives of this study are to identify the potential risk factors in PPUD and to assess the impact of size and site on the overall mortality and in-hospital morbidities.

## Materials and methods

This is a retrospective observational cohort study that was conducted at King Abdulaziz University Hospital (KAUH), Jeddah, Saudi Arabia. We reviewed 750 patients who underwent open exploratory laparotomy (OEL) from 2013 to 2018. Pediatric/Ob/Gyn patients (n=126) and patients who underwent OEL for non-PPUD (n=565), e.g., acute abdomen, cancer, and intestinal obstruction, were excluded. Nine patients who had stomach ulcers caused by gastric cancer were also excluded. A total of 50 patients who had PPUD and underwent OEL with surgical interventions were identified and included in the final analysis. Surgical interventions were at surgeon discretion as either repair, patch, or resection. Demographics and clinical and death data were collected using the electronic health information system of KAUH. Patients were followed up at three, six, and nine months and then yearly in the outpatient clinics of general surgery. At the end of the study, patients were contacted to confirm if they are alive. The primary outcome was defined as a long-term overall mortality. Secondary outcomes were defined as intensive care unit (ICU) admission, ICU length of stay, hospital length of stay, and in-hospital mortality.

Patients were divided into two groups: the small ulcer group if the ulcer is equal to or less than 1 cm in diameter and the large ulcer group if it is larger than 1 cm. Age-adjusted Carlson comorbidity index (ACCI) scores were calculated. For subgroup analysis, patients were categorized into binary variables: small duodenum, large duodenum, small stomach, and large stomach ulcers. Backward stepwise multivariate logistic or linear regression models were built for the study outcomes with a p-value of 0.2. Covariate selection in the models was based on clinical relevance and prior literature. Log-transformation was applied for abnormal distributions variables. Kaplan-Meier curves were constructed to describe the time to death between groups. The log-rank statistics was used to test the overall equality of survival functions between groups. Univariate and multivariate Cox proportional hazard analysis was used to identify independent predictors of overall mortality. All models were selected on least-square or maximum likelihood estimation. P-value < 0.05 was considered as statistically significant. Stata 14 statistical software (StataCorp., College Station, TX, USA) and the R ggplot2 package by Hadley Wickham (2016) were used to analyzed and visualized the data. This study was approved by the Research Ethical Committee at KAUH.

## Results

Patients’ demographics and clinical data

The demographics and clinical data of 50 patients are summarized in Table 1. The mean age was 58.2±17.8, with male predominance of 35 (70%). Of the 50 patients, 21 (42%) were Saudi nationals and 29 (58%) were Non-Saudi nationals, mainly Middle Easterners and Far Easterners nationalities. The majority had presented to the emergency room (ER) with an ACCI score of 1 (30%). PPUD was in the duodenum in 31 (62%) patients. Half (27; 54%) were identified with PPUD larger than 1 cm. Forty-one (41%) patients received general anesthesia (GA) without epidural and 25 (50%) patients received the Graham patch (Table 1). Patients had presented to the ER mostly with abdominal pain (39; 79%), vomiting (24; 48%), and tenderness (20; 40%). Five (10%) patients presented with loss of consciousness (Table 2). Forty (80%) patients underwent abdominal drainage. Sepsis was the second most common complication (15; 30%) (Table 3). Complications included septic shock (15; 30%), multi-organ failure (5; 10%), end-stage renal disease (ESRD) (4; 6%), and heart failure (3; 6%), and these were the common causes of death. The study median follow-up time was 946 days (IQR: 31-1,584 days).

**Table 1:** Descriptive demographic and clinical data Abbreviations: BMI, Body mass index; ACCI, age-adjusted Charlson comorbidity index; GA, general anesthesia

Variables	N = 50
Age, mean ± SD	58.2 ± 17.8
BMI, mean ± SD	25.12 ± 4.4
Male, n (%)	35 (70)
Saudi, n (%)	21 (42)
ACCI score	
1, n (%)	15 (30)
2, n (%)	6 (12)
3, n (%)	7 (14)
4, n (%)	8 (16)
5, n (%)	7 (14)
6, n (%)	5 (10)
>7, n (%)	2 (4)
Site of Ulcer:	
Duodenum, n (%)	31 (62)
Stomach, n (%)	19 (38)
Diameter of ulcer	
Small ≤ 1 cm, n (%)	23 (46)
Large > 1 cm, n (%)	27 (54)
Type of anesthesia	
GA, n (%)	41 (82)
GA with epidural, n (%)	9 (18)
Type of procedure	
Primary repair, n (%)	4 (8)
Graham patch, n (%)	25 (50)
Primary repair and Graham patch, n (%)	16 (32)
Others, n (%)	5 (10)

**Table 2:** Top 10 Presenting clinical manifestations

Clinical manifestation	
Abdominal pain, n (%)	39 (79)
Vomiting, n (%)	24 (48)
Tenderness, n (%)	20 (40)
Nausea, n (%)	14 (28)
Guarding, n (%)	12 (24)
Abdominal distension, n (%)	11 (22)
Hematemesis, n (%)	8 (16)
Constipation, n (%)	7 (14)
Peritonitis, n (%)	6 (12)
Loss of consciousness, n (%)	5 (10)

**Table 3:** Top five post-operative complications

Complications	
Abdominal drainage, n (%)	40 (80)
Sepsis, n (%)	15 (30)
Pneumonia, n (%)	11 (22)
Re-operation, n (%)	8 (16)
Deep surgical infection, n (%)	4 (8)

Primary outcome

**Figure 1:**
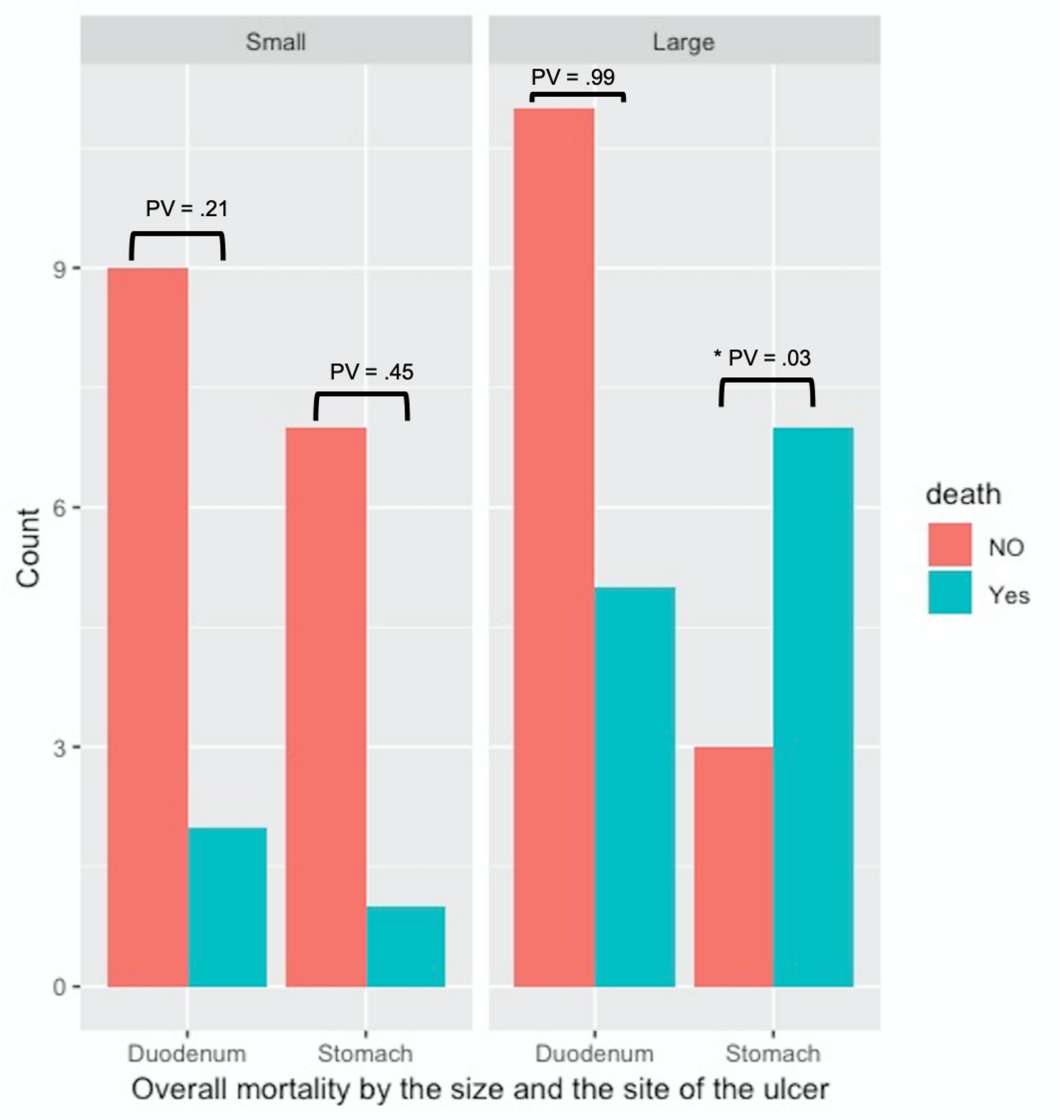
Overall mortality by the size and the site of the ulcer PV: P-value using exact Fisher’s test *PV < 0.05

Over the study period, mortality was 36% (18 patients). The distribution of death and the overall mortality by the site and size of PPUD are illustrated in Figure 1. The overall mortality at 10, 30, and 90 days was 14% (95% CI: 0.06-0.27), 24% (95% CI: 0.14-0.39), and 34% (95% CI: 0.23-0.49), respectively. Using the multivariate Cox regression shown in Table 4, Saudi patients had a 72% decreased risk over time of overall mortality than non-Saudi patients (adjusted HR: 0.28; 95% CI: 0.086-0.89; P=0.03). Patients who had stomach PPUD had 2.23-fold increased risk over time compared to those who had duodenum PPUD (adjusted HR: 2.23; 95% CI: 0.85-5.90; P=0.10). Large PPUD, >1 cm, had a 3.20-fold increased risk over time compared to small PPUD (adjusted HR, 3.20; 95% CI: 1.03-9.86; P=0.40).

**Table 4:** Cox regression for overall mortality Backward stepwise regression with a PV of 0.2. Full model includes BMI, gender, nationality, ACCI score, diameter, type of operation, type of anesthesia, and sites of perforation †Statistically significant Abbreviations: PV, P-value; ACCI, age-adjusted Charlson comorbidity index; PPUD, perforated peptic ulcer disease

Variables	Univariable HR (95% CI)	PV	Multivariable HR (95% CI)	PV
Saudi (ref. non-Saudi)	0.32 (0.11-0.99)	0.05	0.28 (0.086-0.89)	0.03†
ACCI score	1.30 (1.03-1.64)	0.02 †	1.26 (0.97-1.63)	0.08
Stomach PPUD (ref. duodenum)	2.19 (0.89-5.35)	0.11	2.23 (0.85-5.90)	0.10
Large PPUD (ref. small)	2.52 (0.89-7.09)	0.08	3.20 (1.03-9.86)	0.04†

In the subgroup analysis models summarized in Tables 5-8, large stomach PPUD had a 4.22-fold increased risk over time compared to other ulcers, including small stomach, large duodenum, and small duodenum, (adjusted HR; 4.22; 95% CI: 1.41-12.64; P=0.01) (Table 5).

**Table 5:** Subgroups analysis for large stomach PPUD: Cox regression for overall mortality Backward stepwise regression with a PV of 0.2. Full model includes BMI, gender, nationality, ACCI score, diameter, type of operation, type of anesthesia, and sites of perforation † Statistically significant Abbreviations: PV, P-value; ACCI, age-adjusted Charlson comorbidity index; PPUD, perforated peptic ulcer disease

Variables	Univariable HR (CI)	PV	Multivariable HR (CI)	PV
Saudi (ref. non-Saudi)	0.32 (0.11-0.99)	0.05	0.29 (0.10-0.90)	0.03†
ACCI score	1.30 (1.03-1.64)	0.02†	1.22 (0.95-1.58)	0.12
Large stomach PPUD	3.90 (1.50-10.22)	0.01†	4.22 (1.41-12.64)	0.01†

**Table 6:** Subgroups analysis for small stomach PPUD: Cox regression for overall mortality Backward stepwise regression with a PV of 0.2. Full model includes BMI, gender, nationality, ACCI score, diameter, type of operation, type of anesthesia, and sites of perforation † Statistically significant Abbreviations: PV, P-value; ACCI, age-adjusted Charlson comorbidity index; PPUD, perforated peptic ulcer disease

Variables	Univariable HR (CI)	PV	Multivariable HR (CI)	PV
Saudi (ref. non-Saudi)	0.32 (0.11-0.99)	0.05	0.29 (0.10-0.90)	0.03†
ACCI score	1.30 (1.03-1.64)	0.02†	1.33 (1.04-1.71)	0.02†
Small stomach PPUD	0.58 (0.13-2.54)	0.47	0.65 (0.14-3.00)	0.59

**Table 7:** Subgroups analysis for large duodenum PPUD: Cox regression for overall mortality Backward stepwise regression with a PV of 0.2. Full model includes BMI, gender, nationality, ACCI score, diameter, type of operation, type of anesthesia, and sites of perforation † Statistically significant Abbreviations: PV, P-value; ACCI, age-adjusted Charlson comorbidity index; PPUD, perforated peptic ulcer disease

Variables	Univariable HR (CI)	PV	Multivariable HR (CI)	PV
Saudi (ref. non-Saudi)	0.32 (0.11-0.99)	0.05	0.29 (0.10-0.90)	0.03†
ACCI score	1.30 (1.03-1.64)	0.02†	1.36 (1.10-1.74)	0.02†
Large duodenum PPUD	0.87 (0.33-2.33)	0.78	1.11 (0.41-2.99)	0.84

**Table 8:** Subgroups analysis for small duodenum PPUD: stepwise Cox regression for overall mortality Backward stepwise regression with a PV of 0.2. Full model includes BMI, gender, nationality, ACCI score, diameter, type of operation, type of anesthesia, and sites of perforation † Statistically significant Abbreviations: PV, P-value; ACCI, age-adjusted Charlson comorbidity index; PPUD, perforated peptic ulcer disease

Variables	Univariable HR (CI)	PV	Multivariable HR (CI)	PV
Saudi (ref. non-Saudi)	0.32 (0.11-0.99)	0.05	0.29 (0.10-0.90)	0.03†
ACCI score	1.30 (1.03-1.64)	0.02†	1.37 (1.10-1.77)	0.01†
Small duodenum PPUD	0.44 (0.13-1.51)	0.20	0.34 (0.10-1.20)	0.09

Secondary outcomes

Table 9 shows secondary outcomes. A total of 30 (60%) patients were admitted to ICU postoperatively with a median ICU stay of 2 days (IQR: 7 days) and median hospital stay of 13 days (IQR: 16 days). Large PPUD was associated with ICU admission (adjusted OR; 9.36; 95% CI: 1.91-45.77; P=0.01), and patients who received GA with epidural had 90% less admission to ICU compared to GA alone (adjusted OR: 0.10; 95% CI: 0.01-0.82; P=0.03). Female gender and ACCI score were positively linear associated with the increase of log of ICU length of stay. Furthermore, there were increases by 0.87 log ICU length of stay (days) in large PPUD compared to small PPUD when adjusted for other factors (coefficient, 0.87; 95% CI: 0.06-1.69; P=0.03). Similarly, there were increases by 0.67 log hospital length of stay (days) in large PPUD compared to small PPUD when adjusted for other factors (coefficient: 0.67; 95% CI: 0.17-1.14; P=0.01). Saudi had decreased risk of in-hospital mortality compared to non-Saudi by 87% (adjusted OR: 0.13; 95% CI: 0.02-0.90; P=0.04). In-hospital mortality increased by 2.23 folds with every score increase in ACCI score (adjusted OR: 2.23; 95% CI: 1.20-4.16; P=0.01). Both stomach PPUD and large PPUD were associated with in-hospital mortality (adjusted OR: 16.51, 95% CI: 1.70-159.75, P=0.01; (adjusted OR: 11.97, 95% CI: 1.64-87.62, P=0.01, respectively).

**Table 9:** Regressions models for secondary outcomes Backward stepwise regression with a PV of 0.2. Full model includes BMI, gender, nationality, ACCI score, diameter, type of operation, type of anesthesia, and sites of perforation NA, dropped from the final model due to PV more than 0.2 †Statistically significant Abbreviations: ICU, intensive care unit; PV, P-value; ACCI, age-adjusted Charlson comorbidity score; PPUD, perforated peptic ulcer disease

	Multivariable logistic regression for ICU Admission		Multivariable linear regression for log of ICU length of stay		Multivariable logistic regression for in-hospital mortality		Multivariable linear regression for log of hospital length of stay	
Variables	OR (95% CI)	PV	Coefficient (95% CI)	PV	OR (95%CI)	PV	Coefficient (95% CI)	PV
Female (ref. male)	3.80 (0.70-21.73)	0.14	0.88 (0.13-1.62)	0.02†	NA	NA	0.45 (-0.09 to 0.98)	0.10
Saudi (ref. non-Saudi)	NA	NA	NA	NA	0.13 (0.02-0.90)	0.04†	NA	NA
ACCI score	NA	NA	0.31 (0.19-0.51)	0.003†	2.23 (1.20-4.16)	0.01†	0.09 (-0.94 to 0.07)	0.18
GA (ref. GA without epidural)	0.10 (0.01-0.82)	0.03†	NA	NA	0.16 (0.01-1.82)	0.14	NA	NA
Type of operation (ref. primary closure)	NA	NA	-0.20 (-0.47 to 0.08)	0.15	NA	NA	NA	NA
Stomach PPUD (ref. duodenum)	3.90 (0.68-22.14)	0.13	0.72 (-0.04 to 1.48)	0.06	16.51 (1.70-159.75)	0.02†	-0.43 (-0.94 to 0.07)	0.10
Large PPUD (ref. small)	9.36 (1.91-45.77)	0.01†	0.87 (0.06-1.69)	0.03†	11.97 (1.64-87.62)	0.02†	0.67 (0.17-1.14)	0.01†

## Discussion

This study is the first from our region that addresses the association of site and size of PPUD with overall patients’ mortality and in-hospital morbidities. We found that large stomach PPUD increased the risk of overall mortality by 4.22 folds compared to other types of PPUD when we adjust for other related factors. Our data also show that patients with large stomach PPUD underwent complex procedures with or without Graham patch, such as a repair with jejunal patch, gastrostomy, and distal gastrectomy with Roux-en-Y gastrojejunostomy. We did not observe these types of complex surgical procedures in other types of PPUDs, as majority of those patients underwent primary repair with or without Graham patch. This may explain the worse outcomes we have seen in large gastric ulcers in this series of patients of PPUD (Table 1) and may also explain the relatively high 90-day overall mortality of 34% in the studied sample compared to previous studies that reported 30-day mortality rate reaching up to 20% and 90 days mortality rate of up to 30% [1,2,6,7].

We found that the size of perforation is significantly associated with overall mortality in our cohort of patients due to the expected large amount of peritoneal contamination, which will eventually impact on overall patients’ status. Peritonitis was reported in patients with large perforation in other studies [8].

With regard to the secondary outcomes of this study, we found that both stomach PPUD and large PPUD were associated with significant in-hospital mortality. Also, large PPUD and patients who underwent surgery with epidural anesthesia were associated with ICU admission. Moreover, large PPUD, female gender, and ACCI score were associated with increase of ICU length of stay. Interestingly, stomach PPUD was not associated with ICU admission rather than with the ICU length of stay.

With regard to the hospital length of stay stomach PPUD, large PPUD and ACCI score were associated with increase of hospital length of stay. These findings suggest that patients who have a large stomach PPUD might need careful perioperative and postoperative personalized surgical plans as these patients may eventually undergo complicated surgical procedures [3].

Another interesting finding is that Saudi nationals had a decreased risk of overall mortality and in-hospital mortality compared to non-Saudi nationals. This may be attributed to the observations that non-Saudi patients may delay their presentation to medical care due to lack of insurance or financial constraints. Our findings are similar to another Saudi study that investigated Saudi and non-Saudi patients with acute coronary syndrome and showed that non-Saudi patients had a higher risk of mortality versus Saudi patients [9]. Female gender was associated with increase of ICU length of stay but not associated with ICU admission, hospital length of stay, or in-hospital mortality. However, this might be related to the sex biological differences as both sexes are equally treated in our center. ACCI scores were not associated with the ICU admission and hospital length of stay; however, it has done very well in predicting morbidity in our cohort as it was associated with increase of ICU length of stay and in-hospital mortality. Therefore, ACCI score may be helpful in predicting future morbidities in PPUD patients as it does with other emergency general surgery patients [10]. Type of operation did not show any association with findings related to secondary outcomes.

Main complications that lead to mortality in this study are septic shock, multi-organ failure, and ESRD. Septic shock is the major contributing complication (30%). Different reasons are behind this, but the important one is due to late presentation of patients or leakage from the site of closure postoperatively. Sepsis was the main cause of death in the laparoscopic group (4%) [11]. In another study, 5.6% had septicemia due to leak that contributed to mortality [8].

Due to our selection criteria, we did not include patients who underwent a laparoscopic repair for PPUD as it shows no significant difference in hospital length of stay between open and laparoscopic repair postoperatively [6,11].

The main limitation of this study is its small sample size, which is perhaps attributed to a low incidence of PUD and early operative management of the disease in our region, as it was indicated in previous research [12]. Due to this small sample size, some of our findings reached statistical significance with a wide 95% CI. That may affect the generalizability in our statistical models as we could not conduct further subgroup analysis for secondary outcomes.

## Conclusions

Large stomach PPUD is associated with increased overall mortality and morbidity. Patients with large stomach PPUD underwent complex procedures with or without Graham patch, such as a repair with jejunal patch, gastrostomy, and distal gastrectomy with Roux-en-Y gastrojejunostomy. We did not observe these types of complex surgical procedures in other types of PPUD, as majority of those patients underwent primary repair with or without Graham patch. Large stomach PPUD, female gender, and higher ACCI score were associated with increase of ICU length of stay. With regard to the hospital length of stay, large stomach PPUD and higher ACCI score were associated with increase of hospital length of stay. These findings indicate that patients who have a large stomach PPUD might need careful perioperative and postoperative personalized surgical plans as these patients may eventually undergo complicated surgical procedures.
